# Single leg mini squat: an inter-tester reproducibility study of children in the age of 9–10 and 12–14 years presented by various methods of kappa calculation

**DOI:** 10.1186/1471-2474-13-203

**Published:** 2012-10-19

**Authors:** Tina Junge, Sølvi Balsnes, Lisbeth Runge, Birgit Juul-Kristensen, Niels Wedderkopp

**Affiliations:** 1 Institute of Regional Health Services, University of Southern Denmark, Odense, Denmark; 2 Department of Physiotherapy, University College Lillebaelt, Vejle County, Denmark; 3 Institute of Sports Science and Clinical Biomechanics, University of Southern Denmark, Odense, Denmark

**Keywords:** Children, Single Leg Mini Squat, Postural orientation, Reproducibility, Kappa statistics

## Abstract

**Background:**

Multiple studies suggest that reduced postural orientation is a possible risk factor for both patello-femoral joint pain (PFP) and rupture of the anterior cruciate ligament (ACL). In order to prevent PFP and ACL injuries in adolescent athletes, it is necessary to develop simple and predictive screening tests to identify those at high risk. Single Leg Mini Squat (SLMS) is a functional and dynamic real-time screening test, which has shown good validity and reproducibility in evaluation of postural orientation of the knee in an adult population. The aim of this study was to determine the inter-tester reproducibility of SLMS in the age group of 9–10 and 12–14 years by evaluating postural orientation of the ankle, knee, hip and trunk. Further on, this study exemplify the divergence of kappa values when using different methods of calculating kappa for the same dataset.

**Methods:**

A total of 72 non-injured children were included in the study. Postural orientation of the ankle, knee, hip and trunk for both legs was determined by two testers using a four-point scale (ordinal, 0–3). Prevalence, overall agreement as well as four different methods for calculating kappa were evaluated: linear weighted kappa in comparison with un-weighted kappa, prevalence-adjusted bias-adjusted kappa (PABAK) and quadratic weighted kappa.

**Results:**

The linear weighted kappa values ranged between 0.54-0.86 (overall agreement 0.86-0.97), reflecting a moderate to almost perfect agreement. When calculating un-weighted kappa (with and without PABAK) and quadratic weighted kappa, the results spread between 0.46-0.88, 0.50-0.94, and 0.76-0.95, reflecting the various results when using different methods of kappa calculation.

**Conclusions:**

The Single Leg Mini Squat test has moderate to almost perfect reproducibility in children aged 9–10 and 12–14 years when evaluating postural orientation of the ankles, knees, hips and trunk, based on the excellent strength of agreement as presented by linear weighted kappa. The inconsistency in results when using different methods of kappa calculation demonstrated the linear weighted kappa being generally 15% lower than the quadratic weighted values. On average, prevalence-adjusted bias-adjusted kappa increased the un-weighted kappa values by 7% and 12% by children aged 9–10 and 12–14, respectively.

## Background

Patello-femoral joint pain (PFP) and rupture of the anterior cruciate ligament (ACL) are two of the most common complaints and sport injuries among adolescent athletes [1-4]. There has been a clear increase in the number of traumatic ACL injuries among adolescents over the last few years, but the incidence of ACL injuries in children and adolescents is unknown because of the lack of specificity of diagnosis and documentation of skeletal maturity [5]. Multiple studies suggest reduced postural orientation as a possible risk factor in both conditions [1-4,6,7]. Postural orientation, defined as “the ability to maintain an appropriate relationship between the body segments, and between the body and the environment for a task” [8], is suggested to be of great significance to the load on the lower extremities, and especially the frontal plane knee displacement relative to the centerline of the body in functional tasks is considered to determine the quality of the movement [7,9]. Knee displacement is considered a risk factor in terms of future knee injuries [1-3,9,10], but also other components such as displacement of the ankle, hip and trunk, may have an influence on the load of the knee. Therefore, it is recommended that postural orientation should be evaluated over multiple joints when determining displacement of the knee [2,7].

In order to prevent PFP and ACL injuries, screening athletes in the early adolescence is a plausible method to detect and prevent frontal plane knee displacement, why it is necessary to develop simple, reliable and predictive screening tests [2]. Single Leg Mini Squat (SLMS) is a functional and dynamic real-time screening test, which in an adult population has shown good concurrent validity (ROC 0.867, SE 0.082) in relation to 2D analysis, along with an almost perfect inter-tester reproducibility (kappa 0.92, overall agreement 0.96) in the evaluation of frontal plane knee displacement [9]. For children and adolescents at the age of 9–16 years, intra- and inter-tester reproducibility was tested to be moderate (kappa 0.48 and 0.58, respectively; overall agreement 0.76 and 0.79) [10]. In both the aforementioned studies, a nominal scale was applied using un-weighted kappa as statistical method. However, in order to interpret the kappa value it is important to report the prevalence index along with the overall agreement, since both of these values have great impact on kappa [11,12]. By using PABAK (prevalence-adjusted bias-adjusted kappa) [11] kappa can, theoretically, be adjusted for high or low prevalence as well as bias by computing the average prevalence and bias (0.50), as this will give an indication of the likely effects of prevalence and bias.

When using an ordinal scale, weighted kappa is usually recommended, but there is no consensus on which weighting factors to use [11]. This may cause concern about the interpretation of the results, and raises questions about the legitimacy of comparing results between dissimilar methods used to calculate kappa in different studies. In this study, prevalence, overall agreement as well as four different methods for calculating kappa will be evaluated: linear and un-weighted kappa for ordinal data, in comparison with general un-weighted kappa and prevalence-adjusted bias-adjusted kappa (PABAK) and quadratic weighted kappa in nominal data.

Primarily, the aim was to assess the inter-tester reproducibility of the real-time screening test Single Leg Mini Squat within the age group of 9–10 and 12–14 years, by evaluating the postural orientation of the ankle, knee, hip and trunk during the test, presented by linear weighted kappa. The second aim was to exemplify the divergence of kappa values when using different methods of calculating kappa for the same dataset.

## Methods

### Subjects

In total, 74 non-injured children at the age of 9–10 years (n=16 girls, n=21 boys) and 12–14 years (n=20 girls, n=17 boys) representing 3^rd^ and 7^th^ grade from a public school in the Southern part of Denmark were included in this reproducibility study, which is a substudy of the CHAMPS study Denmark [13]. The CHAMPS study Denmark is a longitudinal cohort study, taking place in the period August 2008 - August 2014 in 13 public schools in the municipality of Svendborg, Denmark. The children in 3^rd^ grade performed the test at day 1 and the children in 7^th^ grade at day 2. Children with movement restrictions across the involving joints were excluded. Only two children were excluded from the study due to spasticity and congenital talipes equinovarus, leaving 72 children to be included for the analysis. Participation was voluntary, but all children chose to participate. The children were verbally asked about current back or knee pain prior to the testing. A total of eight children reported minor knee or back pain on the day of examination. They were, however, included in the analysis since the pain was minor and considered to have no impact on their test performance as seen by the testers. None of the children had severe injuries as ACL injuries, but some children may previously have had common sport or leisure time injuries such as sprained ankles. Information regarding prior injuries was obtained by Short Message Service or SMS-tracking, which a new method for registration of musculoskeletal complaints and sport injuries. The children and their parents receive every week an SMS on their cell phone, asking “Has your child had any pain during the past week?” with pain being proxy for musculoskeletal complaints.

Every week, complete lists of children with reported musculoskeletal complaints, are separated from the database, and the parents contacted via telephone by the involved physiotherapists and chiropractors in CHAMPS study Denmark. According to the severity of the musculoskeletal complaint, the character and the extent of the complaint(s), appointments are made for a clinical examination, and every fortnight, the children with need for further unraveling and diagnostics, are examined at their school. Consent forms from parents and the Research Ethics Committee were approved through the CHAMPS study Denmark [13].

### Single Leg mini squat

**Figure 1:**
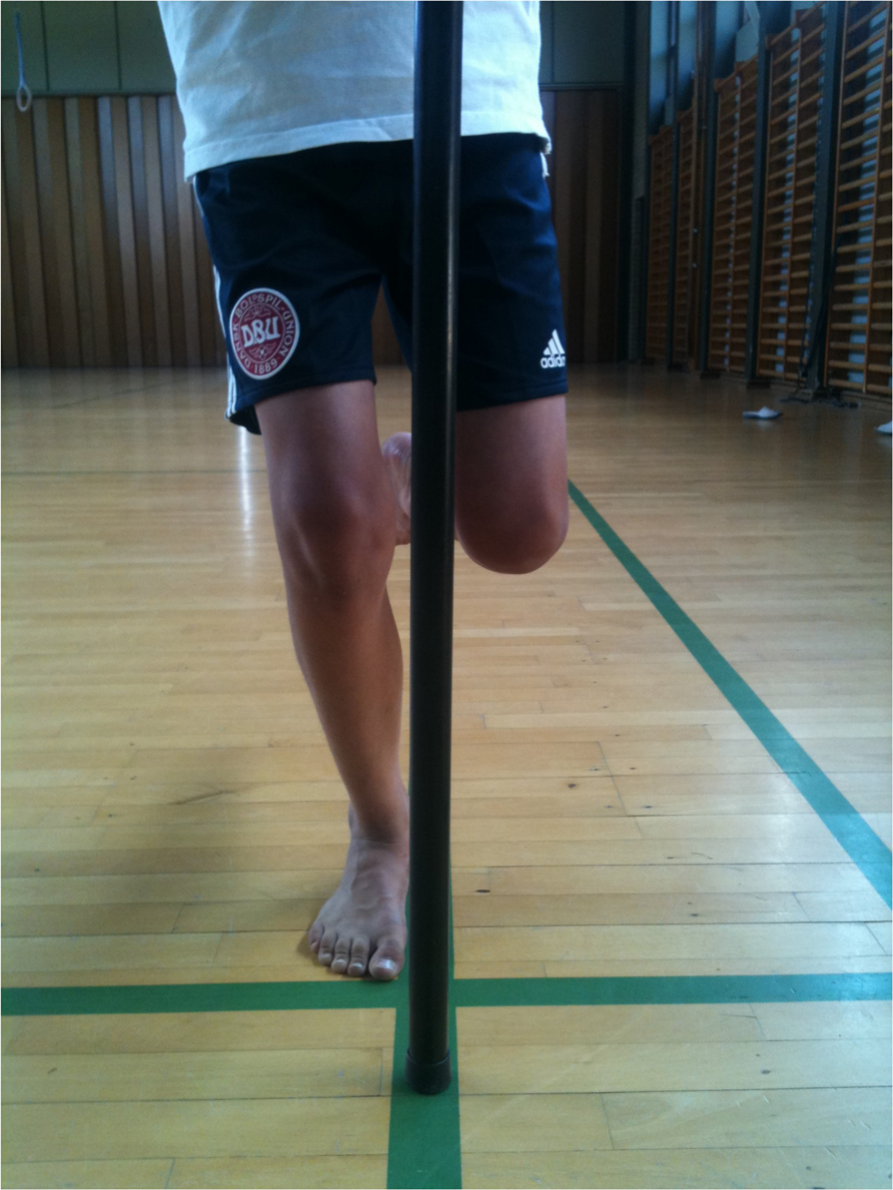
Screening for frontal plane postural orientation of The Single Leg Mini Squat Test.

Standardized instructions for the Single Leg Mini Squat test were given to the child along with a visual demonstration by a physiotherapist performing the test (single legged knee bend). The child were asked to place one foot on a straight line parallel to the length of the foot, with the first toe close to a corresponding horizontal line above the first toe (Figure 1). The index fingers were placed at a bar at navel height for support. Both legs were tested, one at a time, starting on the, by the child, preferred leg.

The instructions were to perform as many knee bends as possible during 30 seconds according to exact instructions, by flexing the knee until the child no longer could see the horizontal line, approximately 50 degrees of knee flexion [10], and then to extend the knee fully again. The trunk had to stay as erect as possible during the test. The child was not informed of the observation of the frontal plane postural orientation, only on the number of knee bends being executed.

The testers (two physiotherapy students) were placed three meters directly in front of the bar. The child was allowed to exercise for one trial of approximately five seconds, where any sagittal plane deviations from the test instructions were corrected by the physiotherapist (e.g. forward bent trunk, too little knee flexion, not extending the knee completely or foot movements).

Frontal plane postural orientation was evaluated by visual observation of the ankle, knee, hip and trunk. The two testers scored each child simultaneously, using a four-point ordinal scale (0 = optimal postural orientation, 1 = possibly reduced postural orientation present, 2 = clearly reduced postural orientation, 3 = so poorly executed that there was no similarity to the instructions given [7], e.g. due to loss of balance). For each of the below described four components the following definitions were used for categorizing frontal plane displacement relative to the centerline of the body: Increased dynamic pronation of the ankle on the leg being tested (ankle), knee medial to second toe (knee), lateral displacement or rotation of hip/pelvis on the stance leg (hip), rotation or lateral displacement of trunk (trunk). The tester scored each child on each component of each leg, based on the general impression of the test performance (i.e. what could be observed during most of the 30 seconds). The standardized instructions and the current Test protocol are presented in the Additional file 1: Appendix 1.

Prior to the study, the two physiotherapy students went, under supervision of an experienced physiotherapist, through a preparation phase, a training phase, an overall agreement phase and a study phase according to the protocol format for reproducibility studies, as suggested by The International Academy of Manual/Musculoskeletal Medicine [12]. The test was standardized before use, including an extensive training phase performed on 100 children aged 10–16, two weeks prior to the study.

### Statistics

For inter-tester reproducibility, a total of eight scores was calculated for each of the 72 children, with a kappa value representing each component for each leg in every child, and also a group mean of each of the two age-groups was derived. Prevalence (P), overall agreement (P_o_) as well as four different values of kappa were calculated: linear weighted kappa (K_w_) in comparison with un-weighted kappa (cut point 0 and 1–3), without and with prevalence-adjusted bias-adjusted kappa (PABAK) [11] and quadratic weighted kappa (K_w_) [14].

Kappa can be adjusted for high or low prevalence by computing the average of cells *a* and *d* in a cross table, substituting this value for the actual values in those cells. Similarly, an adjustment for bias is achieved by substituting the mean of cells *b* and *c* for those actual cell values. The kappa coefficient that results is referred to as PABAK - prevalence-adjusted bias-adjusted kappa [11].

The prevalence index reflects the frequency of positive judged tests, divided by the total number of children in the study population [12]. The overall agreement reflects the percentage of children, in which the testers agree on the outcome or judgment of the test. Overall agreement is calculated by the sum of the number of children in which both testers judge the test positive and in which both testers judge the test negative, divided by the total number of children in the study [12]. When applying linear or quadratic weighted kappa, the overall agreement is calculated according to the weights chosen.

Kappa values were classified as <0.0 = poor, 0.0-0.20 = slight, 0.21-0.40 = fair, 0.41-0.60 = moderate, 0.61-0.80 = substantial, 0.81-1.00 = almost perfect [15].

All calculations and statistical analysis were conducted in STATA (version 12.0) (Statacorp, College Station, Texas, USA).

## Results

The Single Leg Mini Squat test has moderate to excellent reproducibility for children in the age of 9–10, representing 3^rd^ grade and 12–14 years, representing 7^th^ grade, with linear weighted kappa ranging from 0.54 to 0.86. The linear weighted kappa is generally 15% lower than the quadratic weighted values, ranging from 0.76 to 0.95. On average, PABAK increases the un-weighted kappa values by 7% and 12% in 3^rd^ and 7^th^ grade, respectively.

The lowest linear weighted kappa value in both grades is 0.59 for the children in 3^rd^ grade and 0.54 for the children in 7^th^ grade (left and right knee respectively), both representing a moderate strength of agreement. The corresponding overall agreement (P_o_ 0.87 and 0.86) is relatively high.

The evaluation of trunk displacement shows the highest kappa, while the knees are the most difficult body components to judge (Table 1). Generally, for linear weighted kappa, 6% of the kappa values are almost perfect, 75% are substantial and 19% of kappa values are moderate, along with a generally high overall agreement in both grades.

**Table 1:** **Weighted kappa (K**_**w**_**) and overall agreement (P**_**o**_**) using linear weights, frontal plane postural orientation, 3**^**rd **^**and 7**^**th **^**grade (n= 72)**

**3**^**rd **^**grade**	**K**_**w**_	**P**_**o**_	**7**^**th **^**grade**	**K**_**w**_	**P**_**o**_
**Right ankle**	0.71	0.92	**Right ankle**	0.60	0.92
**- knee**	0.68	0.89	**- knee**	0.54	0.86
**- hip**	0.80	0.93	**- hip**	0.61	0.89
**- trunk**	0.86	0.97	**- trunk**	0.73	0.96
**Left ankle**	0.67	0.92	**Left ankle**	0.61	0.92
**- knee**	0.59	0.87	**- knee**	0.66	0.92
**- hip**	0.70	0.91	**- hip**	0.70	0.92
**- trunk**	0.77	0.94	**- trunk**	0.77	0.97

Table 2 (right leg of the children in 7^th^ grade by linear weighting), illustrates as an example the actual scores that underlies the kappa statistics. In Table 3 (right knee of the children in 7^th^ grade by linear weighting) the precision of the scores is exemplified, as one tester scores 5 children ´2´, while the other tester scores 11 children ´2´, demonstrating that one tester finds more than twice as many children with a clear knee displacement compared to the other tester. The large discrepancy as described in Table 3 is, however, only observed in 3/16 of the cross tables, indicating that this is not a general tendency.

**Table 2:** **Two testers actual scores for frontal plane postural orientation, right leg, 7**^**th **^**grade (n= 37)**

**Right leg 7**^**th **^**grade**	**0**	**1**	**2**	**3**
**Ankle**	24/31	9/4	3/1	1/1
**Knee**	21/17	10/8	5/11	1/1
**Hip**	12/16	20/12	4/8	1/1
**Trunk**	30/34	5/1	1/1	1/1
				Total 37/37

**Table 3:** **Inter-tester reproducibility, right knee, 7**^**th **^**grade (n= 37)**

	**0**	**1**	**2**	**3**	**Total**
**0**	15	2	0	0	17
**1**	5	2	1	0	8
**2**	1	6	4	0	**11**
**3**	0	0	0	1	1
**Total**	21	10	**5**	1	37

Bold numbers symbolize inter-tester differences.

With respect to statistical methods of agreement, kappa values are highest in quadratic weighting (0.76-0.95, 3^rd^ grade; 0.69-0.91, 7^th^ grade), and lowest in un-weighted kappa (0.52-0.88 (3^rd^ grade; 0.47-0.63, 7^th^ grade) (Tables 4 and 5). On average, PABAK increases the un-weighted kappa values by 7% and 12% in 3^rd^ and 7^th^ grade, respectively; while kappa increases 17% and 22% in average for each grade when using quadratic compared with linear weighting. Overall agreement also varies with the different kappa methods applied; for the right knee of the children in the 7^th^ grade the linear weighted overall agreement is 0.86, the quadratic weighted increases to 0.95 and the un-weighted kappa is 0.59.

**Table 4:** **Prevalence and four types of different kappa statistics for calculating frontal plane postural orientation, 3**^**rd **^**grade (n= 35)**

**3**^**rd **^**grade**	**Prevalence (score 1–3)**	**Kappa**	**PABAK**	**Linear weighted kappa**	**Quadratic weighted kappa**
**Right ankle**	0.33	0.71	0.72	0.71	0.85
**- knee**	0.42	0.52	0.50	0.68	0.82
**- hip**	0.39	0.72	0.72	0.80	0.87
**- trunk**	0.08	0.88	0.94	0.86	0.95
**Left ankle**	0.28	0.58	0.61	0.67	0.81
**- knee**	0.42	0.54	0.54	0.59	0.76
**- hip**	0.17	0.46	0.61	0.70	0.80
**- trunk**	0.19	0.78	0.83	0.77	0.85

**Table 5:** **Prevalence and four types of different kappa statistics for calculating frontal plane postural orientation, 7**^**th **^**grade (n= 37)**

**7**^**th **^**grade**	**Prevalence (score 1–3)**	**Kappa**	**PABAK**	**Linear weighted kappa**	**Quadratic weighted kappa**
**Right ankle**	0.13	0.63	0.62	0.60	0.76
**- knee**	0.37	0.58	0.58	0.54	0.69
**- hip**	0.47	0.63	0.63	0.61	0.69
**- trunk**	0.08	0.56	0.78	0.73	0.86
**Left ankle**	0.26	0.53	0.56	0.61	0.80
**- knee**	0.34	0.47	0.46	0.66	0.82
**- hip**	0.47	0.47	0.46	0.70	0.83
**- trunk**	0.08	0.54	0.78	0.77	0.91

When looking at the mean values of children in 3^rd^ and 7^th^ grade by linear weighting the mean kappa value for both grades is 0.67, while for quadratic weighting, kappa values are above 0.80 for all four components. For both un-weighted kappa values (without and with PABAK) only 6% and 13% of the components are above kappa 0.80.

## Discussion

The findings of this study indicate that the Single Leg Mini Squat test has moderate to excellent reproducibility for children in the age of 9–10, representing 3^rd^ grade and 12–14 years, representing 7^th^ grade, tested by 2 inexperienced physiotherapists, with linear weighted kappa ranging from 0.54 to 0.86 being only 15% lower than the quadratic weighted values, ranging from 0.76 to 0.95. The lowest kappa values were observed at children in 7^th^ grade on the knee component with a linear weighted kappa of 0.54. Further on, this study indicates that very different results are presented depending on the type of kappa chosen as statistical method.

### Discussion of aim 1

The linear weighted kappa found in the current study varies from 0.54 to 0.86 (P 0.08 – 0.47, P_o_ 0.86 - 0.97) depending on the component to be evaluated with the knee component as the most challenging. A previous study on adults evaluating the reproducibility of SLMS in adults found a higher kappa (kappa 0.92, Po 0.96) when using a nominal scale assessing postural orientation of the knee solely [9], while this study evaluated postural orientation of multiple components and with an ordinal scale. The number of possible scores (0–3) and the number of components (4) may have influenced the judgment of postural orientation and thereby the results, considering that an ordinal scale may be less accurate than a nominal scale, due to a higher risk for the two testers to disagree [9,11]. The protocol for the Single Leg Mini Squat test for children was accomplished according to the description of Ortqvist [10], but with the scoring system of Trulsson [7] in order to try to differentiate the degree of displacements. The latter concept was originally developed for a test performed 5 times only and in a much slower pace, why it in this study can be difficult for the tester to manage to observe the described components on four different regions (ankle, knee, hip, trunk) and score on four different points (0–3) in a faster pace during 30 seconds. Another example of a real-time multi-component scoring system for evaluating frontal plane postural orientation in a jump-landing task is the Landing Error Scoring System - Real Time (LESS-RT) [16]. 5 components with 2–3 scoring possibilities are scored over 2 trials of the jump-landing task with an additional trial to allow the tester to observe all 5 jump-landing characteristics. For athletes (18–23 years) the LESS-RT has high inter-rater reliability (ICC 0.79, 95% CI 0.64-0.88), evaluated by experienced athletic trainers. Yet, this system has not been tested on children or adolescents and still lacks predictive evidence for identifying individuals who are at high risk for injury.

When assessing the reproducibility of SLMS in a child population, a moderate inter-tester reproducibility (kappa 0.57) and an overall agreement of 0.79 were observed [10]. Dichotomizing data from the right knee of the 7^th^ grade to a nominal scale (0=negative test, 1-3=positive test), the current study obtained, with a prevalence of 0.37, a kappa value identical to the kappa presented by Ortqvist et al. (2010) (kappa 0.58, overall agreement 0.59 vs. 0.79). Ortqvist et al. concludes that the test clinically is useful in a pediatric population, based upon the relatively high overall agreement. However, this is in discrepancy with Landis & Koch, as overall agreement does not take into account the occurrence of agreement by statistical chance [15]. To determine a given test’s reproducibility, the most comprehensive presentation is reported to include the prevalence, the overall agreement and the kappa value in combination [11,12].

Kappa for the knee component is generally lower for children in the 7^th^ grade compared with children in the 3^rd^ grade. Possible explanations for this could be a high or low prevalence index, or that the children in 7^th^ grade perform the test faster and hence produce a higher number of knee bends (median 22 vs. 17) making it more challenging to determine the score.

The trunk was the component with the highest kappa values. An explanation for this phenomenon could be that trunk displacement is easier to observe visually compared with displacement of the knee. The current protocol may not be thorough enough regarding determination of score, why it is advisable for future studies to standardize the test more detailed with emphasis on the knee component.

Prior to the study, the two physiotherapy students went, under supervision of an experienced physiotherapist, through standardization of the test in an extensive training phase performed on a large number of children in order to minimize bias and increase overall agreement. With a strict, standardized protocol and a thorough training phase, clinical experience ought to be less important, which some studies [9,17] assessing movement quality indicates.

### Discussion of aim 2

The authors of this study have chosen to interpret their results using linear weighted kappa (Table 1). However, it would have been possible to choose quadratic weighted kappa, which would have increased the reproducibility for SLMS (see Tables 4 and 5). Quadratic weighted kappa is often used for the purpose of comparing results with Intraclass Correlation Coefficient (ICC) [18]. However the concern, the quadratic weighted kappa method may give a too positive picture of the reproducibility of this screening test with an equal distance between scoring categories, as quadratic weights increases with the number of categories, whereas linearly weights varies much less with the number of categories [11]. As well as the kappa, the overall agreement increases depending on the type of kappa chosen as statistical method, with a divergence as much as 61% from un-weighted kappa to quadratic weighted kappa in the example of the right knee for the children in the 7^th^ grade. Clinically, one must consider the application of the results of the quadratic weighted kappa, demonstrating an almost perfect reproducibility along with a very high overall agreement, which might provide several false-positive outcomes.

When interpreting the cross table (Table 3) as an example, it reveals that one or both of the testers obviously have over- or underestimated the displacement of the right knee of the children in the 7^th^ grade. This illustrates why kappa values never can be used independently to evaluate the ability of a given test, but needs to be interpreted with the prevalence index and optimum with the content of cross tables presented, in addition to the overall agreement, in order to understand the full potential of a test’s reproducibility [18]. The amount of misclassifications becomes clearer by observing the numbers in a cross table compared with only knowing the kappa value [18].

Tables 4 and 5 illustrates how different methods of calculating kappa may influence the final result and therefore the reported reproducibility. The results vary from moderate to excellent strength of agreement within the same component, which is a serious inconsistency, considering that they are calculated on the same dataset. The concern here is that there are no formal guidelines available as to when one should use which weighting values. Depending on what seems natural in the given context, it is even possible to develop one’s own weighting scale [11,14] which is a considerable limitation for comparison of results, unless the weighting values are described and explained.

Another concern regarding this study and kappa statistics in general, is the benchmarking for an acceptable kappa value. When interpreting the kappa values according to Landis & Koch [15], 60% of the quadratic and linear weighted single component results would be substantial and 34% almost perfect. However, classification for interpreting the kappa values varies, and the benchmark for an acceptable kappa value, classified as intermediate to good, is according to Fleiss 0.40, and for an excellent kappa value 0.75 [19]. This means that 75% of the kappa values in this study would be intermediate to good and 25% excellent. The question is, how high the inter-tester reproducibility coefficient should be for the extent of agreement to be considered good enough, and since the choice of classification scale and benchmark inevitably will be arbitrary, one should always interpret kappa in relation to the prevalence, the overall agreement and the bias [11].

The limitations of this study are the amount of components to be evaluated in a relative short time interval, and using an overall score without determining exactly when, during the 30 seconds, to evaluate on which component, concerning the influence of muscle fatigue on the performance. It may therefore be too demanding to be accurate when evaluating postural orientation on several components in a real-time test. A potential solution may be to consider the use of 2D video analysis with the option to observe one body part at a time, and to use the video facilities as slow motion and repetition as in other similar studies [6,9].

In this study, the contrasts between children was minimal, as only non-injured children and only few children with minor pain participated, which can have affected the testers likelihood of scoring ´2´ or ´3´, as injured children most probably would have had higher scores. A study population consisting of injured and non-injured children could have affected the prevalence and might have made it more obviously for the testers when to score a ´2´ or ´3´. In clinical practice it is important to be able to screen children with potentially injury risks, which was one of the reasons for performing a reproducibility study in a study population that is normally seen in clinical practice. Using PABAK, as presented in the current study, may solve one of the statistical problems with the small group contrast*.* Screening tests with differentiated scores on an ordinal scale may also be more similar to clinical practice than screening tests on a nominal scale, why the necessity of evaluating the reliability as well as the predictive validity of screening tests becomes clear.

The strengths of this study are the high number of children included and thus the amount of information collected. Previous studies have only examined one component (the knee) at a time, while this study indicates that SLMS has potential as a screening test evaluating postural orientation of several components. The method is fast and easy to administer for clinical use and requires no equipment. The test was standardized before use, with an extensive training period performed on 100 children.

If the SLMS test is a predictor of complaints and sport injuries in children and adolescents, it could be used as a screening tool, thus targeting interventions at those children and adolescents with displacement of ankle, knee, hip or trunk components during the test. However, it is necessary to test concurrent and predictive validity in children and adolescents in relation to PFP and ACL injuries before such interventions are relevant.

## Conclusions

The Single Leg Mini Squat test has moderate to almost perfect reproducibility in children aged 9–10 and 12–14 years when evaluating postural orientation of the ankles, knees, hips and trunk, based on the excellent strength of agreement as presented by linear weighted kappa.

Further, this study clearly shows the inconsistency in results when using different methods of kappa calculation demonstrating the linear weighted kappa being generally 15% lower than the quadratic weighted values. On average, prevalence-adjusted bias-adjusted kappa increased the un-weighted kappa values by 7% and 12% by children aged 9–10 and 12–14, respectively.

## Abbreviations

PFP: Patello-femoral joint pain; ACL: Anterior cruciate ligament; SLMS: Single Leg Mini Squat; PABAK: Prevalence-adjusted bias-adjusted kappa.

## Competing interests

The authors declare that they have no competing interests.

## Authors’ contributions

TJ, LR and BJK contributed to the design of the study. TJ, SB and LR collected the data. TJ, SB and LR performed the data management. TJ, LR and NW performed the data analysis and were in charge of data interpretation. TJ and SB wrote the manuscript. All authors participated in data interpretation and contributed to manuscript revision. All authors read and approved the final version.

## Pre-publication history

The pre-publication history for this paper can be accessed here:

http://www.biomedcentral.com/1471-2474/13/203/prepub

## Supplementary Material

Additional file 1 **Appendix 1.**Protocol Single Leg Mini Squat. Click here for file

## References

[B1] Griffin LY Albohm MJ Arendt EA Understanding and preventing noncontact anterior cruciate ligament injuries: a review of the Hunt Valley II meeting, January 2005 Am J Sports Med 2006 34 9 1512 1532 10.1177/0363546506286866 16905673

[B2] Hewett TE Ford KR Hoogenboom BJ Myer GD Understanding and preventing injuries: Current biomechanical and epidemiologic considerations - update 2010 North Am J Sports Physical Therapy 2010 5 4 234 251 PMC3096145 21655382

[B3] Ireland ML Wilson J Ballantyne BT Davis IM Hip strength in females with and without patellofemoral pain J Orthop Sports Phys Ther 2003 33 11 671 676 14669962 10.2519/jospt.2003.33.11.671

[B4] Willson JD Davis IS Lower extremity mechanics of females with and without patellofemoral pain across activities with progressively greater task demands Clin Biomechanics 2008 23 2 203 211 10.1016/j.clinbiomech.2007.08.025 17942202

[B5] Paletta GA Jr Special considerations, Anterior cruciate ligament reconstruction in the skeletally immature Orthop Clin North Am 2003 34 1 65 77 10.1016/S0030-5898(02)00067-6 12735202

[B6] Stensrud S Myklebust G Kristianslund E Correlation between two-dimensional video analysis and subjective assessment in evaluating knee control among elite female team handball players Br J Sports Med 2011 45 7 589 595 10.1136/bjsm.2010.078287 21148569

[B7] Trulsson A Garwicz M Ageberg E Postural orientation in subjects with anterior cruciate ligament injury: development and first evaluation of a new observational test battery Knee surgery, sports traumatology, arthroscopy: official journal of the ESSKA 2010 18 6 814 823 10.1007/s00167-009-0959-x 19851755

[B8] Horak FB Macpherson JM Shepard J, Rowell L Postural orientation and equilibrium Handbook of physiology 1996 section 12 New York: Oxford University 255 292

[B9] Ageberg E Bennell KL Hunt MA Validity and inter-rater reliability of medio-lateral knee motion observed during a single-limb mini squat BMC Musculoskelet Disord 2010 11 265 10.1186/1471-2474-11-265 21080945 PMC2998461

[B10] Ortqvist M Mostrom EB Roos EM Reliability and reference values of two clinical measurements of dynamic and static knee position in healthy children Knee surgery, sports traumatology, arthroscopy: official journal of the ESSKA 2011 19 12 2060 2066 10.1007/s00167-011-1542-9 21584720

[B11] Sim J Wright CC The Kappa Statistic in Reliability Studies: Use, Interpretation, and Sample Size Requirements Phys Ther 2005 85 3 257 268 15733050

[B12] Patijn J Remvig L Reproducibility and Validity Protocol Formats for Diagnostic Procedures in Manual/Musculoskeletal Medicine 2007 23–36, 57–75 Available from: http://www.fimm-online.com

[B13] Wedderkopp N The CHAMPS study Denmark 2008 Available from: http://www.sdu.dk/om_sdu/institutter_centre/rich/forskning/forskningsprojekter/svendborg+projektet

[B14] Cohen J Weighted kappa: Nominal scale agreement with provision for scaled disagreement or partial credit Phychological Bulletin 1968 70 4 213 220 10.1037/h0026256 19673146

[B15] Landis JT Koch GG The Measurement of Observer Agreement for Categorical Data Biometrics 1977 33 1 159 174 10.2307/2529310 843571

[B16] Padua DA Reliability of the landing error scoring system-real time, a clinical assessment tool of jump-landing biomechanics J Sport Rehabil 2011 20 2 145 156 21576707 10.1123/jsr.20.2.145

[B17] Enoch F Inter-examiner reproducibility of tests for lumbar motor control BMC Musculoskelet Disord 2011 12 114 10.1186/1471-2474-12-114 21612650 PMC3116485

[B18] De Vet HCW Terwee CB Mokkink LB Knol DL Reliability Measurement in Medicine 2011 New York: Cambridge University Press 96 149

[B19] Fleiss JL Statistical Methods for Rates and Proportions 1981 2 New York: Wiley

