# Supplementary material for: Single leg mini squat: an inter-tester reproducibility study of children in the age of 9–10 and 12–14 years presented by various methods of kappa calculation
Source: BMC Musculoskelet Disord. 2012 Oct 19;13:203. doi: 10.1186/1471-2474-13-203 (PMC3560108; doi:10.1186/1471-2474-13-203)
Supplement: Additional file 1 — Appendix 1.Protocol Single Leg Mini Squat. [file 1471-2474-13-203-S1.pdf]

Protocol Single Leg Mini Squat

Purpose of the test

In the test Single Leg Mini Squat the number of single legged knee bends during 30 seconds is registered. Frontal plane postural orientation is evaluated by visual observation of the ankle, knee, hip and trunk.

Setup

A “T” is marked on the floor with adhesive tape. The child is asked to place one foot on a straight line parallel to the length of the foot, with the first toe close to a corresponding horizontal line above the first toe. The index fingers are placed upon a music stand at navel height for support. Both legs must be tested, one at a time.

Test performance

During 30 seconds the child performs as many knee bends as possible according to exact instructions, by flexing the knee until the child no longer can see the horizontal line, approximately 50 degrees of knee flexion, and then to extend the knee fully again. The trunk has to stay as erect as possible during the test. The child has to wear short trousers and T-shirt.

Instructions

”Bend the knee, until you can not see the horizontal line any more, and then extend the knee fully again. When you have a sense of how much you must bend, you have to look straight out. Perform as many knee bends as possible exactly as I told you. I will stop you after 30 seconds”.

Practice trial

The child has one practice trial of 5-10 seconds of each leg prior to the tests.

Test

The child has one test trial to perform as many knee bends as possible during 30 seconds, according to exact instructions.

Error

- The child places the opposite foot at the floor several times despite corrections.
- The child consecutive do not bend the knee sufficient despite corrections.
- The child consecutive do not extend the knee sufficient despite corrections.
- The child consecutive moves the foot on the standing leg despite corrections.
- The child consecutive bends the trunk forward despite corrections.

Score

- The number of single legged knee bends during 30 seconds is registered for each leg.
- Frontal plane postural orientation is evaluated by visual observation of the dominant image of the ankle, knee, hip and trunk for right and left leg. A letter and a number must be scored for each component, starting from the ankle and up.

1) Ancle: Increased dynamic pronation of the ankle on the leg being tested, compared to a two-legged stance

2) Knee: Medial displacement of knee to second toe

3) Hip: Lateral displacement or rotation of hip/pelvis on the stance leg

4) Trunk: Rotation or lateral displacement of trunk

0: Optimal postural orientation

1: Possibly reduced postural orientation present (an experienced tester could see the displacement)

2: Displacement clearly present (an inexperienced person could see the displacement)

3: The child performed very poorly (no similarity to the instructions given e.g. consecutive errors despite corrections or if the child could not complete the 30 seconds)
